# A Human iPSC-Derived Neuron and Glioma Culture Model Reveals Increased Neuronal Excitability and Loss of GABAergic Inhibition

**DOI:** 10.21203/rs.3.rs-11002745/v1

**Published:** 2026-10-12

**Authors:** Hannah Haile, Brianna Pereira, Jacqueline Niu, Karen Conboy, Sandra Leskinen, Angeliki Mela, Athanassios Dovas, August Kahle, Abby L. Brand, Ashwin Viswanathan, Nelson Humala, Aayushi Mahajan, Tasneem Meem, Roland A. Scott, Anthony J. Tang, Misha Amini, Wisdom Yevudza, Christopher D. Makinson, Jeffrey N. Bruce, Catherine Schevon, Henry Colecraft, Peter Canoll, Brian J. A. Gill

## Abstract

Gliomas engage in bidirectional interactions with neural circuits, driving hyperexcitability, disruption of excitatory-inhibitory balance, and tumor-associated seizures. Human *in vitro* models that enable functional interrogation of tumor-induced changes in glutamatergic-GABAergic neuronal crosstalk remain limited. We established a defined human induced pluripotent stem cell (iPSC)-derived culture comprising of glutamatergic neurons, GABAergic neurons, and astrocytes with and without glioma cells. Network maturation and function were characterized using immunocytochemistry, multi-electrode array recordings, and whole-cell patch-clamp electrophysiology. Neuronal cultures developed mature synaptic architecture, spontaneous coordinated network activity, and suppression of neuronal firing following treatment with the GABA-A agonist muscimol, demonstrating functional GABAergic signaling. Glioma incorporation increased spike rate, increased coordinated network activity, and reversed the response to muscimol from suppression to elevation of neuronal firing, indicating disrupted GABAergic signaling. Whole-cell patch-clamp recordings demonstrated increased intrinsic excitability of glutamatergic neurons following glioma co-culture, characterized by increased action potential firing frequency and increased action potential amplitude. This human iPSC-derived glutamatergic–GABAergic neuron model recapitulates key features of tumor-induced neuronal dysfunction while enabling complementary network- and single-cell analyses. The model provides a relevant, scalable, and accessible system for mechanistic investigation of bidirectional neuron–glioma interactions and preclinical evaluation of therapies targeting tumor-induced neuronal dysfunction.

